# Genetics of Aldosterone-Producing Adenoma in Korean Patients

**DOI:** 10.1371/journal.pone.0147590

**Published:** 2016-01-25

**Authors:** A. Ram Hong, Jung Hee Kim, Young Shin Song, Kyu Eun Lee, Soo Hyun Seo, Moon-Woo Seong, Chan Soo Shin, Sang Wan Kim, Seong Yeon Kim

**Affiliations:** 1 Department of Internal Medicine, Seoul National University College of Medicine, Seoul, Republic of Korea; 2 Department of Surgery, Seoul National University College of Medicine, Seoul, Republic of Korea; 3 Department of Laboratory Medicine, Seoul National University College of Medicine, Seoul, Republic of Korea; 4 Department of Internal Medicine, Seoul Metropolitan Government Boramae Medical Center, Seoul, Republic of Korea; University of Heidelberg, GERMANY

## Abstract

**Objectives:**

Recently, somatic mutations in *KCNJ5*, *ATP1A1*, *ATP2B3*, and *CACNA1D* genes were found to be associated with the pathogenesis of aldosterone-producing adenoma (APA). This study aimed to investigate the prevalence of somatic mutations in *KCNJ5*, *ATP1A1*, *ATP2B3*, and *CACNA1D* and examine the correlations between these mutations and the clinical and biochemical characteristics in Korean patients with APA.

**Methods:**

We performed targeted gene sequencing in 66 patients with APA to detect somatic mutations in these genes.

**Results:**

Somatic *KCNJ5* mutations were found in 47 (71.2%) of the 66 patients with APA (31 cases of p.G151R and 16 cases of p.L168R); these two mutations were mutually exclusive. Somatic mutations in the *ATP1A1*, *ATP2B3*, and *CACNA1D* genes were not observed. Somatic *KCNJ5* mutations were more prevalent in female patients (66% versus 36.8%, respectively; *P* = 0.030). Moreover, patients with *KCNJ5* mutations comprised a significantly higher proportion of patients younger than 35 years of age (19.1% versus 0%, respectively; *P* = 0.040). There were no significant differences in pre-operative blood pressure, plasma aldosterone, serum potassium, lateralization index, and adenoma size according to mutational status. Patients with *KCNJ5* mutations were less likely to need antihypertensive medications after adrenalectomy compared with those without mutation (36.2% versus 63.2%; *P* = 0.045).

**Conclusions:**

The present study demonstrated the high prevalence of somatic *KCNJ5* mutations in Korean patients with APA. Carriers of somatic *KCNJ5* mutations were more likely to be female. Early diagnosis and better therapeutic outcomes were associated with somatic *KCNJ5* mutations in APA.

## Introduction

Primary aldosteronism (PA) is the most common cause of secondary hypertension and accounts for 10% or more of hypertension cases. PA is also associated with a high prevalence of cardiovascular events and target organ damage [1, 2]. However, the molecular mechanisms of PA are not completely understood.

Mutations in the *KCNJ5* gene, which encodes G-protein-activated inward-rectifying K^+^ channel 4, cause familial hyperaldosteronism type III and sporadic aldosterone-producing adenoma (APA) [3, 4]. These mutations are located near or within the selective filter of the K^+^ channel, resulting in increased Na^+^ conductance through loss of ion selective permeability [5]. Subsequent cell membrane depolarization activates voltage-gated Ca^2+^ channels, thereby stimulating the expression of the *CYP11B2* gene, which encodes aldosterone synthase [6].

The prevalence of *KCNJ5* mutations in sporadic APA varies depending on ethnicity. In Caucasians, *KCNJ5* mutations are found in approximately 40% of patients, whereas in Eastern Asians, about 70% of APA patients carry this mutation [7, 8]. Intriguingly, somatic *KCNJ5* mutations are more prevalent in female patients with APA [7, 9]. Some studies have also reported more severe phenotypes of APA, including younger age at diagnosis, higher plasma aldosterone, and lower serum potassium levels, in patients with somatic mutations in *KCNJ5* [10–12].

Recently, somatic mutations in the *ATP1A1* gene (which encodes Na^+^K^+^-ATPase), the *ATP2B3* gene (which encodes Ca^2+^-ATPase), and the *CACNA1AD* gene (which encodes the voltage-gated Ca^2+^ channel, L type) were also detected in patients with sporadic APA. However, the clinical implications of these mutations remain unknown. Moreover, no previous studies have investigated somatic mutations in Korean individuals with APA.

Therefore, in this study, we aimed to explore the prevalence of somatic mutations in *KCNJ5*, *ATP1A1*, *ATP2B3*, and *CACNA1AD* in Korean patients with sporadic APA and to evaluate the clinical and biochemical characteristics associated with these mutations.

## Materials and Methods

### Patients

The study was approved by the Institutional Review Board of Seoul National University Hospital (SNUH) and was conducted according to the Declaration of Helsinki. All subjects enrolled in this study were Korean and gave written informed consent.

Patients with APA who underwent unilateral adrenalectomy between January 2004 and October 2014 were recruited at SNUH. PA was diagnosed in accordance with the current Endocrine Society Guideline [13]. A final diagnosis of APA was considered when all of the following criteria were satisfied: 1) histological identification of adenoma; 2) normalization of hypokalemia, if present; 3) cure or improvement of hypertension; 4) normalization of the aldosterone to renin ratio; and 5) suppression of aldosterone under saline infusion [14]. We initially recruited 90 patients with APA whose formalin-fixed, paraffin-embedded (FFPE) tissue samples were stored. We excluded eight patients because of insufficient FFPE samples and 16 patients due to inadequate quality of DNA for comprehensive mutational analysis. Thus, the present analysis was performed in a total of 66 patients with APA.

### Clinical and biochemical parameters

We collected information on clinical and biochemical parameters, such as age at diagnosis, body mass index (BMI), gender, systolic/diastolic blood pressure, antihypertensive medications, plasma aldosterone, plasma renin activity, serum potassium, glomerular filtration rate, lateralization index at adrenal vein sampling (AVS), and adenoma size by retrieving data from medical records. Postoperative blood pressure and biochemical parameters were measured at 3 months after the operation. Plasma aldosterone concentrations were measured by RIA using the SPAC-S aldosterone kit (TFB, Inc.). The intra- and interassay coefficients of variation (CVs) were 4.7% and 4.5%, respectively. Plasma renin activity was measured using the Renin RIA beads (TFB, Inc., Tokyo, Japan) before 2011 and using a PRA RIA kit (TFB, Inc.) since 2011. The intra- and interassay CVs were 3.8% and 6.7%, respectively. Measurement of adenoma size was based on pathological tissue specimens.

### DNA isolation, amplification, and sequencing of the *KCNJ5*, *ATP1A1*, *ATP2B3*, and *CACNA1D* genes

Genomic DNA was extracted from 10-μm-thick sections of 10% neutral FFPE adenoma tissue samples using a QIAamp DNA Mini Kit (Qiagen, Hilden, Germany).

Targeted gene sequencing was performed as previously described [15]. Ten nanograms of DNA was used for multiplex PCR of covered coding regions in the *KCNJ5*, *ATP1A1*, *ATP2B3*, and *CACNA1D* genes (Ion AmpliSeq panel; Life Technologies, Grand Island, NY, USA). Fragment libraries were constructed by DNA fragmentation, barcode and adaptor ligation, and library amplification using an Ion DNA Barcoding kit (Life Technologies) according to the manufacturer’s instructions. The size distribution of the DNA fragments was analyzed on an Agilent Bioanalyzer using a High Sensitivity Kit (Agilent, Santa Clara, CA, USA). Template preparation, emulsion PCR, and ion sphere particle (ISP) enrichment were performed using an Ion Xpress Template kit (Life Technologies) according to the manufacturer’s instructions. The ISPs were loaded onto a P1 chip and sequenced using an Ion P1 sequencing kit (Life Technologies).

### Bioinformatic analysis

After successful sequencing, the raw data were processed using Ion Torrent platform-specific pipeline software (Torrent Suite v4.4). The pipeline was used to generate sequence leads filtered based on this software quality control, and to remove poor signal reads. Reads assembling and variant calling were performed using Torrent Suite with a plug-in program (variant caller v4.4). For downstream analysis, variants with minimum coverage of 500 reads containing at least 5% of mutant reads were selected. Variant calls were further analyzed using internally developed software that allowed variant filtering and annotation using refGene in UCSC, COSMIC v.67, dbSNP build 138, and ExAC. The *in silico* prediction algorithms SIFT, PolyPhen-2, Mutation Taster, and PROVEAN were used to predict potential deterioration on protein function. To minimize false positives, variants were finally filtered with a normal population variant database, the Korean Personal Genomes Project (http://opengenome.net/)[16].

### Statistical analysis

Data are expressed as the mean ± standard deviation (SD), median (25th and 75th percentiles), or *n* (%). Normally distributed continuous variables were analyzed using *t*-tests, and skewed variables were analyzed using Mann-Whitney tests. Categorical variables were compared with chi-square tests. All statistical analyses were performed using PASW SPSS for Windows (Version 21, SPSS Inc., Chicago, IL, USA). *P* values of less than 0.05 were considered statistically significant.

## Results

Somatic *KCNJ5* mutations were found in 47 (71.2%) patients (Table 1). In patients with *KCNJ5* mutations, p.G151R (c.451G>A or c.415G>C) and p.L168R (c.503T>G) substitutions accounted for 66.0% (31/47) and 34.0% (16/47), respectively. The two mutations were mutually exclusive; therefore, each patient with a somatic *KCNJ5* mutation had either p.G151R or p.L168R. Of the 31 cases with p.G151R, 21 carried c.451G>A, and the remaining 10 carried c.451G>C. Somatic mutations in *ATP1A1*, *ATP2B3*, and *CACNA1D* were not found in the present study.

**Table 1:** Prevalence of somatic *KCNJ5* mutation across different countries.

City (Country)	Number of APA	KCNJ5 mutated, *n* (%)	G151R (%)	L168R (%)	Other mutations	Reference
Seoul (Korea)	66	47 (71.2)	66.0	34.0	-	Our study
Maebashi (Japan)	23	15 (65.0)	80.0	20.0	-	4
Yokohama (Japan)	108	75 (69.4)	60.5	39.5	T158A (*n* = 1), E145Q (*n* = 2)	17
Shanghai (China)	168	129 (76.8)	51.9	46.5	T158A (*n* = 1), T148-T149insR (*n* = 1)	12
Beijing (China)	114	86 (75.4)	50.0	45.3	T158A (*n* = 2)	19
Taipei (Taiwan)	148	91 (61.5)	49.5	45.1	T158A (*n* = 1), I157del (*n* = 1)	18
Paris (France)	199	74 (37.2)	62.2	37.8	-	9
München (Germany)	93	32 (34.4)	56.3	43.7	-	9
Berlin (Germany)	23	11 (47.8)	45.5	54.5	-	9
Torino (Italy)	73	29 (39.7)	65.5	27.6	T158A (*n* = 1), W126R (*n* = 1)	9
Padova A (Italy)	37	14 (37.8)	57.1	42.9	-	9
Padova B (Italy)	66	15 (22.7)	66.7	33.3	-	10
Padova C (Italy)	37	14 (37.8)	57.1	42.9	-	10
Rome (Italy)	43	6 (14.0)	33.3	66.7	-	10
Cambridge (United Kingdom)	46	20 (43.5)	55.0	40.0	I157del (*n* = 1)	18

APA, aldosterone-producing adenoma.

The correlations between pre-operative clinical and biochemical parameters and *KCNJ5* mutational status are summarized in Table 2. A higher proportion of patients with somatic *KCNJ5* mutations was female (66% versus 36.8%, respectively; *P* = 0.030). Age at diagnosis of APA was similar in patients with and without *KCNJ5* mutations; however, patients with *KCNJ5* mutations comprised a significantly higher proportion of patients younger than 35 years of age (19.1% versus 0%, respectively; *P* = 0.040). BMI was lower in patients with *KCNJ5* mutations, which may be attributed to the female predominance in patients with *KCNJ5* mutations. There were no significant differences in baseline blood pressure, number of antihypertensive medications, glomerular filtration rate, and localization of adenoma between patients with and without *KCNJ5* mutations. Pre-operative serum potassium, plasma aldosterone level, aldosterone to renin ratio, lateralization index at AVS, and adenoma size were similar between the two groups. When the clinical and biochemical characteristics of APA were compared between patients with and without somatic *KCNJ5* mutations after adjusting for age, gender, and BMI by analysis of covariance (ANCOVA), no significant differences were observed between the two groups.

**Table 2:** The correlation between somatic *KCNJ5* mutational status and preoperative clinical and biochemical parameters.

Variable	*KCNJ5* wild-type APA (*n* = 19)	*KCNJ5* mutated APA (*n* = 47)	*P*	Adjusted P*
Age at diagnosis (years)	48.5 ± 9.6	46.6 ± 12.7	0.562	-
Age at diagnosis <35 years, *n* (%)	0 (0.0)	9 (19.1)	0.040	-
Female, *n* (%)	7 (36.8)	31 (66.0)	0.030	-
Body mass index (kg/m^2^)	26.2 ± 3.4	23.6 ± 3.3	0.007	-
SBP (mmHg)	152 ± 13	145 ± 18	0.150	0.264
DBP (mmHg)	95 ± 8	93 ± 15	0.632	0.844
Preoperative number of anti-hypertensive medications	3 (1, 4)	2 (1, 3)	0.105	0.389
Glomerular filtration rate (mL/min/1.73m^2^)	80.0 ± 23.4	89.3 ± 28.3	0.221	0.821
Preoperative lowest serum potassium level (mmol/L)	2.9 (2.6, 3.0)	2.8 (2.5, 3.1)	0.842	0.877
Preoperative PAC (ng/dL)	48.2 (33.0, 55.2)	41.3 (31.7, 52.5)	0.395	0.607
Preoperative PRA (ng/mL·hr)	0.10 (0.10, 0.10)	0.10 (0.10, 0.19)	0.352	0.358
Preoperative ARR (ng/dL)/(ng/mL·hr)	432 (271, 536)	358 (182, 489)	0.315	0.407
Lateralization index at adrenal vein sampling	16.6 (5.2, 25.2)	21.4 (9.4, 38.7)	0.169	0.249
Largest tumor size (cm)	1.8 (1.2, 2.1)	1.5 (1.2, 2.0)	0.793	0.462

Data are presented as mean ± SD, median (25th, 75th), or *n* (%).

SBP, systolic blood pressure; DBP, diastolic blood pressure; PAC, plasma aldosterone concentration; PRA, plasma renin activity; ARR, aldosterone to renin ratio.

* *P* values were calculated after adjusting for age, gender, and body mass index.

We further explored the response to adrenalectomy according to mutational status (Table 3). No associations were observed between *KCNJ5* mutations and postoperative serum potassium, plasma aldosterone level, and blood pressure. However, the proportion of patients taking antihypertensive medications after adrenalectomy was significantly lower in patients with *KCNJ5* mutation (36.2% versus 63.2%, respectively; *P* = 0.045).

**Table 3:** The correlation between somatic *KCNJ5* mutational status and postoperative clinical and biochemical parameters.

Variable	*KCNJ5* wild-type APA (*n* = 19)	*KCNJ5* mutated APA (*n* = 47)	*P*
Postoperative SBP (mmHg)	131 ± 15	129 ± 14	0.542
Postoperative DBP (mmHg)	85 ± 12	84 ± 11	0.985
Postoperative serum potassium level (mmol/L)	4.5 (4.2, 5.1)	4.5 (4.3, 4.8)	0.758
Postoperative PAC (ng/dL)	9.0 (6.8, 15.9)	11.7 (7.3, 15.5)	0.530
Postoperative PRA (ng/mL·hr)	1.38 (0.30, 2.34)	1.00 (0.50, 3.17)	0.867
Postoperative ARR (ng/dL)/(ng/mL·hr)	6.9 (4.2, 25.9)	8.8 (4.3, 20.2)	0.874
Postoperative number of patients taking anti-hypertensive medications	12 (63.2)	17 (36.2)	0.045

Data are presented as mean ± SD, median (25th, 75th), or *n* (%).

SBP, systolic blood pressure; DBP, diastolic blood pressure; PAC, plasma aldosterone concentration; PRA, plasma renin activity; ARR, aldosterone to renin ratio.

We performed *in silico* analysis using SIFT, PolyPhen-2, Mutation Taster, and PROVEAN algorithms to predict potential changes in protein function. When the SIFT algorithm was used, p.G151R and p.L168R mutations were predicted to affect protein function, with scores of 0 and 0.001, respectively, indicating that the mutations were damaging. Both p.G151R and p.L168R mutations were predicted as probably damaging using PolyPhen-2, disease causing using Mutation Taster, and damaging by PROVEAN. There were no significant differences in clinical or biochemical characteristics between patients with p.G151R and p.L168R mutations.

## Discussion

The present study is the first to investigate somatic mutations in the *KCNJ5*, *ATP1A1*, *ATP2B3*, and *CACNA1AD* genes in Korean patients with APA. In this study, 71.2% of patients had somatic *KCNJ5* mutations; this high prevalence of *KCNJ5* mutations is consistent with previous reports from two Japanese populations, two Chinese populations, and one Taiwanese population in which *KCNJ5* mutations were present in 65%, 69%, 77%, 75.4%, and 59.5% of patients, respectively [4, 12, 17–19]. On the other hand, the overall prevalence of *KCNJ5* mutations in Caucasians is approximately 40%. It is unclear why the prevalence of somatic *KCNJ5* mutations are higher in Asian countries than elsewhere; however, this may be attributed to the increased prevalence of PA and dietary habits, particularly higher sodium intake, in Asian populations [4, 20]. Somatic mutations in the *ATP1A1*, *ATP2B3*, and *CACNA1D* genes were not observed in the present study.

Somatic mutations in *KCNJ5* were more common in female than in male patient in our patient population, consistent with the results of previous studies [6, 9, 10, 21–23]. Interestingly, this female predominance of somatic *KCNJ5* mutation has been consistently reported not only in Caucasians but also in Eastern Asians [12, 17]. The underlying mechanisms mediating this gender difference in *KCNJ5* mutations are still unknown. However, evaluation of the *KCNK3*^-/-^ mice model may provide insights into these mechanisms. For example, only female *KCNK3*^-/-^ mice exhibited hyperaldosteronism, and testosterone-treated *KCNK3*^-/-^ females showed normal aldosterone levels, implying an androgen-protective effect in male mice [6]. In addition, our study showed that patients with somatic mutations in *KCNJ5* were likely to be diagnosed earlier than those without mutations. The association between *KCNJ5* mutations and younger age at diagnosis has been observed in some previous reports, but not in all studies [4, 10]. The present study supported the possible association between the presence of somatic *KCNJ5* mutations and early detection of hyperaldosteronism because of a more severe APA phenotype.

We did not observe correlations between *KCNJ5* mutations and adenoma size or disease severity factors, such as lower serum potassium or higher plasma aldosterone level. More severe phenotypes of APA have been observed in patients with *KCNJ5* mutations in some studies [10, 12, 24] but not in others [3, 4, 23]. In addition, patients harboring *KCNJ5* mutations were found to have higher lateralization indexes at AVS in a previous report [25]; however, this association was not observed in our current study. In a recently published meta-analysis from 1636 patients with APA, larger tumor size and higher plasma aldosterone levels were associated with somatic *KCNJ5* mutations, but serum potassium and blood pressure levels showed no significant association with *KCNJ5* mutation [20]. The potential association between *KCNJ5* mutations and disease severity remains to be clarified.

Interestingly, somatic mutations in *KCNJ5* were also associated with postoperative treatment for blood pressure in the present study, similar to results in the Taiwanese population [18]. However, serum potassium and aldosterone levels were similar, regardless of the presence of *KCNJ5* mutations. Although there has been no consensus on the correlation between somatic *KCNJ5* mutations and postoperative response, early diagnosis in patients with *KCNJ5* mutations may reduce the disease duration and decrease irreversible cardiovascular changes.

There were no somatic mutations in the *ATP1A1*, *ATP2B3*, and *CACNA1D* genes in our study. These mutations are responsible for increases in aldosterone production via activation of the Ca^2+^ signaling pathway and have been considered to be exclusive of *KCNJ5* mutations [23, 26]. In studies in Caucasians, these mutations have been shown to be present in less than 10% of patients with APA [9, 23]. In two Chinese populations and one Taiwanese population, the prevalence rates of *ATP1A1*, *ATP2B3*, and *CACNA1D* mutations were lower than in Caucasians, approximately less than 2%, respectively [12, 18, 19]. Although the precise mechanisms underlying the differences in the prevalence rates of the *ATP1A1*, *ATP2B3*, and *CACNA1D* mutations are unclear, ethnic factors as well as the small sample size may be related to the low prevalence of somatic mutations in these genes in the present study.

The present study has several limitations. First, this study was conducted with samples collected at a single tertiary referral center; hence, the sample size was not large enough to represent the entire population of Korean patients with APA. Because of the small sample size and lack of correlations between the *KCNJ5* mutation and disease-specific parameters related to APA except management of hypertension, we could not strongly recommend early diagnosis of APA based on the *KCNJ5* mutation status. Additionally, we did not evaluate germline *KCNJ5* mutations. Although the association with APA and the presence of somatic *KCNJ5* mutation has been consistently reported worldwide, germline *KCNJ5* mutations have been observed in approximately 6% of patients with sporadic APA [8]. Therefore, even though sample size was small, we could not rule out the possibility of germline mutation in the *KCNJ5* gene in the present study.

In summary, in this report, we presented the first Korean study of somatic mutations in patients with APA. We observed a high prevalence of somatic *KCNJ5* mutations in patients with APA; however, there were no somatic mutations in the *ATP1A1*, *ATP2B3*, and *CACNA1D* genes. Somatic *KCNJ5* mutations were more common in female and patients diagnosed at a younger age. Additionally, *KCNJ5* mutations were associated with better outcomes in postoperative blood pressure in our patient population. We anticipate that the results of this study may guide future mutational studies of APA in Korean patients.
